# Intertendinous epidermoid cyst of the forearm

**DOI:** 10.1080/23320885.2018.1564314

**Published:** 2019-01-28

**Authors:** Motomu Suito, Takeshi Kitazawa, Kazuhiro Tsunekawa, Masato Shiba, Tsuneko Ikeda

**Affiliations:** a Department of Plastic and Reconstructive Surgery, Matsunami General Hospital, Hashima-gun, Japan;; b Department of Plastic and Reconstructive Surgery, Shinshu University School of Medicine, Matsumoto, Japan;; c Department of Human Pathology, Matsunami General Hospital, Hashima-gun, Japan

**Keywords:** Epidermoid cyst, epidermal cyst, epidermal inclusion cyst, giant, deep fascia, forearm

## Abstract

As epidermoid cysts generally originate from hair follicle infundibulum, they appear as intradermal or subcutaneous tumors and are very rare in locations away from the skin. Here, we report a rare case of intertendinous epidermoid cyst of the forearm in a 68-year-old man that was treated surgically.

## Introduction

Epidermoid cysts are common benign tumors presenting anywhere over the body [1], and are generally detected as intradermal or subcutaneous tumors. Here, we report a rare case of an epidermoid cyst located under the deep fascia in the forearm, which was removed successfully.

## Case presentation

**Figure 1.**
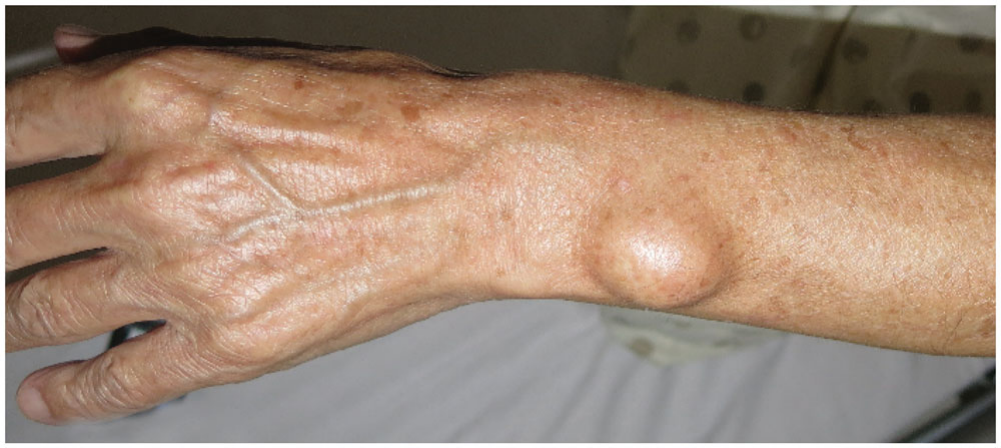
Preoperative physical examination. The swelling was elastic and hard. There was no scar and no central punctum.

A 68-year-old right-handed Japanese man presented to our hospital complaining of a gradually growing swelling in his left forearm. He had noticed it for 3 years but did not remember any causal events. It was elastic, hard, and had poor mobility, without tenderness, and there were no findings suggesting its adhesion to the overlying skin. The patient showed neither motor impairment nor sensory disturbance of the hand and fingers. (Figure 1)

**Figure 2.**
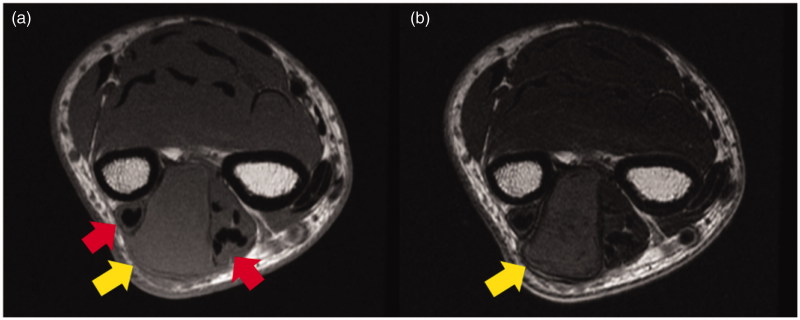
Preoperative MRI findings. (a) MRI (T1-weighed image): the mass was slightly hyperintense compared to the muscle and pushing outward against the extensor tendons (arrows). (b) MRI (T2-weighed image): the mass was slightly hyperintense compared to the muscle (arrows).

Magnetic resonance imaging (MRI) showed a unilocular and well-defined cystic mass (31 × 22 × 15 mm) in the forearm. It showed a slightly high intensity on both T1-weighted and T2-weighed images and was pushing out the extensor tendons (Figure 2). Based on these findings, the differential diagnosis included soft tissue sarcoma, fibroma, hematoma, high-protein fluid, etc. Although fine-needle aspiration was performed to obtain material for histological analysis, nothing was suctioned from the tumor.

**Figure 3.**
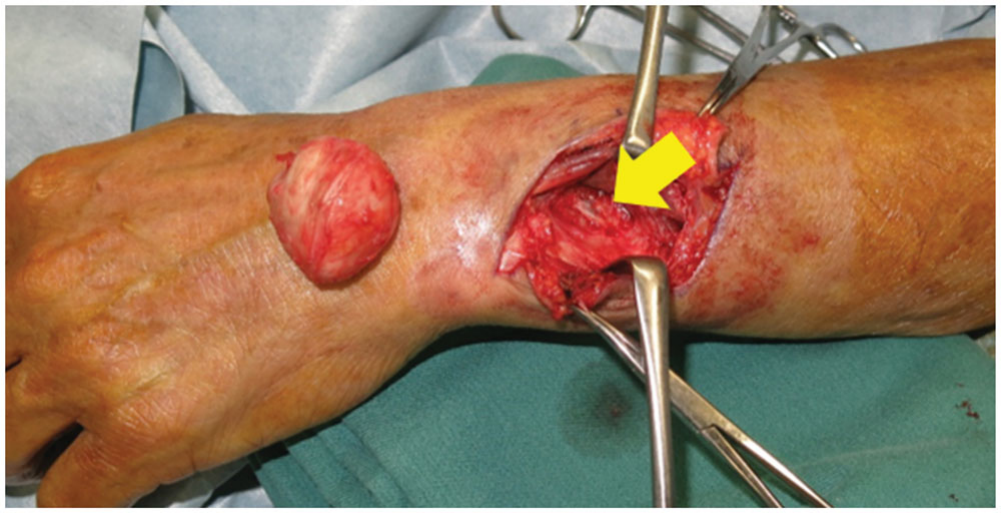
Intraoperative photograph (arrow: antebrachial interosseous membrane). The cystic mass was covered by a thick white capsule and its bottom was adjacent to the antebrachial interosseous membrane. There was no scarring around the fascia.

Excisional biopsy was performed under general anesthesia with a tourniquet. The mass was detected under the deep fascia and was not connected to the skin. It was covered with a thick white capsule and its bottom was adjacent to the antebrachial interosseous membrane (Figure 3). As the cyst did not adhere to the surrounding structures and the boundary was clear, it was easily removed. White creamy material was seen within the capsule.

**Figure 4.**
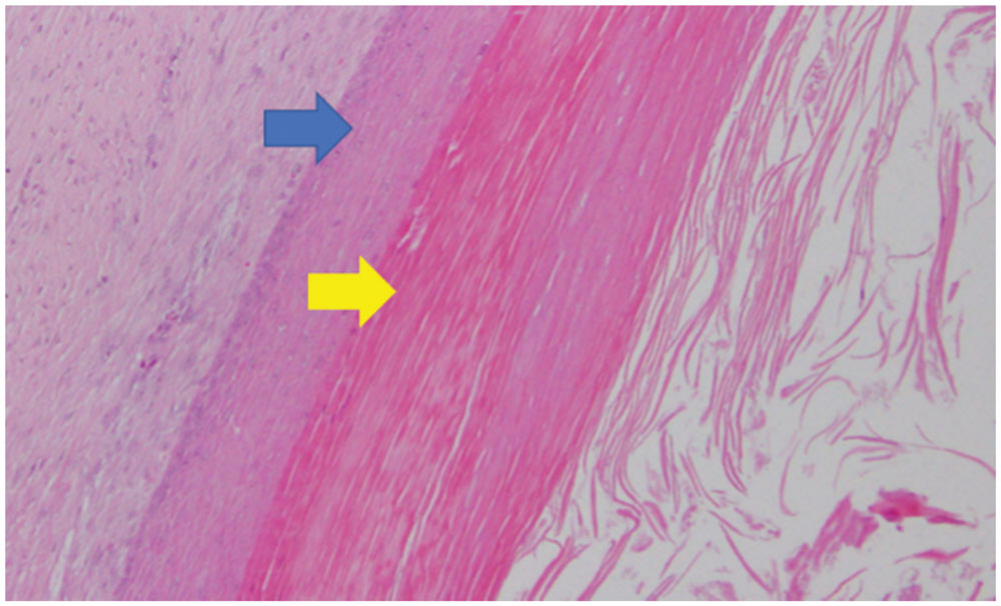
Histopathological findings (hematoxylin-eosin staining, original magnification × 100) upper arrow: squamous epithelium lining, lower arrow: keratin collections. The cyst wall was composed of stratified squamous epithelium with abundant keratin. There were no hair tissue structures or atypical cells.

Histopathological examination showed that the cyst wall was composed of stratified squamous epithelium with abundant keratin. The definite diagnosis was epidermoid cyst without malignancy (Figure 4).

There have been no complications during a 6-month follow-up after surgery.

## Discussion

Epidermoid cysts are common benign intradermal or subcutaneous tumors presenting anywhere over the body, including glabrous sites [1]. The majority of epidermoid cysts encountered in general practice are derived from hair follicle infundibulum and are confined to the subcutaneous tissue. They are slow growing, rarely reach a size >5 cm [2], and are strongly suspected in cases with a central punctum of the external skin [3]. Therefore, it is necessary to resect not only the cyst but also the contiguous skin.

In the present case, however, the cyst was located under the deep fascia, and neither radiological examination nor intraoperative findings showed continuity between the cyst and the skin. Similar epidermoid cysts presenting deep in the body have been reported in the presacral region [4], spleen [5], cecum [6], deep in the neck [7], and deep abdominal wall [8]. They are thought to be congenital, derived from ectodermal implantation during embryogenesis [9]. Especially among those of congenital origin, a rare subtype of epidermoid cysts called giant epidermoid cysts are known to grow rapidly to 5 cm or more, occur considerably deep in the body, and lack a central punctum [3,10].

On the other hand, acquired epidermoid cysts have been reported to be derived from hair follicle infundibulum, trauma, surgery, spinal anesthetic injection, etc. [11–13]. They are thought to be due to the implantation of epithelial cells into deep dermal tissues [9]. Human papillomavirus infection also leads to the development of epidermoid cysts from eccrine ducts in glabrous sites, such as the plantar and sole of the foot [14].

The imaging features of epidermoid cysts vary according to the degree of cyst maturation, concentration, and amount of keratin [4]. Ultrasound sonography (US) and MRI are useful for the diagnosis of epidermoid cysts [2]. Kim et al. [15] suggested that the following findings strengthen preoperative diagnosis. On the US, mildly echogenic masses with occasional linear anechoic and/or echogenic reflections, hypoechoic rim, and no Doppler flow. On MRI, T2 hyperintense masses, possibly with low-signal-intensity debris, and thin rim enhancement on contrast-enhanced T1-weighed images. In the present case, however, the mass showed slightly high both T1 and T2 signal intensity on MRI. These findings were different from the imaging features mentioned above, which also made diagnosis difficult in the present case. Therefore, MRI alone did not lead to a diagnosis. On reflection, it would have been beneficial to have performed the US in this case. It is difficult to diagnose epidermoid cysts based on the results of preoperative imaging examination. However, even if the lesions do not exhibit the “typical” image of epidermoid cysts, the accuracy of diagnosis can be increased by keeping in mind the possibility of epidermoid cysts.

In the present case, the mass was detected under the deep fascia and was not attached to the skin. The patient had no history of trauma or surgery, and we found no evidence of such possible caused on physical examination. Based on these findings, a congenital origin was the most likely explanation for the presence of the epidermoid cyst under the deep fascia in this case.

Epidermoid cysts can present at any location and any depth in the body. It is important not to exclude epidermoid cysts from diagnosis based only on a lack of continuity with the skin. Both US and MRI should be performed preoperatively.

## Conclusion

We encountered a rare case of epidermoid cyst presenting under the deep fascia. For masses in deep parts of the body, it is important not to exclude epidermoid cysts from the possible diagnoses.
